# Clinical Routine Application of the Second-generation Neuroendocrine Markers ISL1, INSM1, and Secretagogin in Neuroendocrine Neoplasia: Staining Outcomes and Potential Clues for Determining Tumor Origin

**DOI:** 10.1007/s12022-020-09645-y

**Published:** 2020-08-19

**Authors:** Carl Christofer Juhlin, Jan Zedenius, Anders Höög

**Affiliations:** 1 Department of Oncology-Pathology, Karolinska Institutet, Stockholm, Sweden; 2 Department of Pathology and Cytology, Karolinska University Hospital, Stockholm, Sweden; 3 Department of Breast, Endocrine Tumors and Sarcoma, Karolinska University Hospital, Stockholm, Sweden; 4 Department of Molecular Medicine and Surgery, Karolinska Institutet, Solna, Sweden

**Keywords:** Neuroendocrine neoplasia, Chromogranin A, Synaptophysin, ISL1, INSM1, Secretagogin

## Abstract

Neuroendocrine neoplasms (NENs) have traditionally been identified via expression of proteins associated to the regulation of secretory vesicles and granules. We report the clinical usage of the “second-generation” proteins ISL LIM homeobox 1 (ISL1), INSM transcriptional repressor 1 (INSM1), and secretagogin (SECG) as immunohistochemical markers of neuroendocrine differentiation since their introduction in clinical routine and compare the results with the established proteins chromogranin A (CGA) and synaptophysin (SYP). In total, 161 tumors, including 139 NENs and 22 “non-NENs” (unrelated tumors with an initial suspicion of NEN), were informatively stained for ISL1, and subsets were also interrogated for INSM1 and/or SECG. Diffuse or focal positive immunoreactivity was noted for ISL1 in 91/139 NENs (65%) and in 6/22 (27%) non-NENs, for INSM1 in 76/85 NENs (89%) and in 2/5 (40%) non-NENs, and for SECG in 49 out of 64 NENs (77%) and in 0/5 non-NENs (0%). Generally, ISL1, INSM1, and SECG exhibited sensitivities in line with or slightly below that of CGA and SYP—largely attributable to tissue-specific patterns regarding tumoral origin. Moreover, for pancreatic and small intestinal NENs, the two largest subgroups, ISL1 staining results were consistent irrespectively of tumor source and WHO grade. We verify previously suggested immunohistochemical schemes of neuroendocrine markers of first- and second-generations to facilitate the diagnostic process for NENs and confirm that the second-generation neuroendocrine markers display tissue-specific patterns. We therefore recommend their implementation in tertiary endocrine pathology centers, not least to aid in the identification of primary tumors when analyzing metastases.

**Electronic supplementary material:**

The online version of this article (10.1007/s12022-020-09645-y) contains supplementary material, which is available to authorized users.

## Introduction

Staining of proteins associated to the protein machinery regulating the secretory granules of neuroendocrine cells has revolutionized endocrine pathology, starting with the Grimelius silver stain in the 1960s, followed by immunohistochemical analyses targeting chromogranin A (CGA) and synaptophysin (SYP). The diagnostic arsenal has since expanded, with ISL LIM homeobox 1 (ISL1), INSM transcriptional repressor 1 (INSM1), and secretagogin (SECG) to name a few novel markers exhibiting high sensitivity and specificity for neuroendocrine neoplasms (NENs).

ISL1 was originally found to be expressed in neuroendocrine cells as well as in neurons of the peripheral nervous system [1]. Since then, ISL1 has been established as a master DNA-binding transcriptional activator that is essential for pancreatic exo- and endocrine differentiation, and ISL1 has been found to bind directly to the insulin gene promoter and regulate insulin gene expression [2–4]. Today, we know that ISL1 also carries ubiquitous roles in embryological processes, such as regulation of the BMP, WNT, and FGF signaling pathways in the development of external genitalia [5]. From a diagnostic perspective, ISL1 immunoreactivity was originally reported as a specific marker of pancreatic NENs (Pan-NENs), and the authors suggested that ISL1 immunohistochemistry could be employed to determinate the primary site of a metastatic NEN, in which a positive stain strongly would indicate a pancreatic origin [6]. In a succeeding publication, ISL1 immunoreactivity was also noted in duodenal NENs [7], followed by a report demonstrating ISL1 expression in NENs developed from extra-pancreatic locations, including all examined rectal NENs, and a majority of duodenal NENs, and in subsets of colonic NENs, appendiceal NENs, and small intestinal NENs (SI-NENs) [8]. Subsequently, ISL1 expression has also been reported as a highly sensitive marker also for additional neuroendocrine neoplasms, such as Merkel cell carcinomas, pheochromocytomas, paragangliomas, and medullary thyroid carcinomas [9]. As of this, the diagnostic role of ISL1 immunoreactivity has shifted somewhat, from an original viewpoint suggesting Pan-NEN specificity to that of a general neuroendocrine marker. Even so, ISL1 immunoreactivity could still indicate tissue-specific origin regarding metastatic well-differentiated NENs, in which the 3-marker panel TTF1, CDX2, and ISL1 could separate rectal and Pan-NENs from lung and SI-NENs [10]. ISL1 immunoreactivity has also been reported in poorly differentiated neuroendocrine carcinomas (NECs), suggesting that the marker could be of value in determining neuroendocrine differentiation also in dedifferentiated forms of the disease [9, 11].

INSM1 was originally described as an insulinoma-associated transcription factor that plays a key role in neurogenesis and neuroendocrine cell differentiation during embryonic development [12, 13] and has since been thoroughly investigated for its role as a marker of neuroendocrine differentiation. Studies have found that INSM1 is a reliable marker for neuroendocrine differentiation in various tumors, including Merkel cell carcinomas and NENs of the lung, gynecological tract, prostate, and head-neck region s[14–21]. Notably, for pulmonary NECs, INSM1 has been proposed as a more sensitive marker than conventional neuroendocrine markers of the first generation [22].

SECG is a 32 kDa protein with calcium-binding properties discovered some 20 years ago, and initial analyses did pinpoint a selective expression of Langerhans cells in pancreatic tissues as opposed to the exocrine compartment [23–25]. Subsequent analyses have revealed rather neuroendocrine-specific expression patterns, and the marker has been suggested to be a complement to the first-line marker CGA, as SECG was consistently positive also in colorectal NENs negative for CGA [26, 27].

At the Karolinska University Hospital, we have routinely used CGA and SYP immunohistochemistry as screening markers of neuroendocrine differentiation. Other markers such as CD56 and chromogranin B have been used much more periodically at our institution, as the former marker is fairly promiscuous in terms of tumor specificity, and the latter has a limited clinical usage in our experience—except for prognostication of pheochromocytomas and paragangliomas (PPGLs) [28, 29]. We introduced ISL1 immunohistochemistry in 2017 after successful validation of antibodies for clinical routine purposes, followed by INSM1 later the same year, while SECG was included in the diagnostic arsenal in late 2018. We here present the staining results of these newly established markers in a large NEN cohort, validating several previous findings and highlighting novel associations of potential diagnostic value. As most previous studies regarding these second-generation neuroendocrine markers are retrospective in nature, we wanted to share our experiences using a prospective staining setting in a high-volume referral center.

## Materials and Methods

### Tumor Cohort

An overview of the tumor cases included in this study is available in Table 1 and Supplementary Table 1. The starting date of the study origins from the first case stained for ISL1 in the clinical setting (February 2017), and continued until December 2019, when the collection phase was closed and staining information and final diagnoses for all cases were summarized. All tumors were diagnosed using the most recent WHO criteria at the time of diagnosis, and all NENs were graded through a Ki-67 proliferation index. In all, 161 tumors, including 139 NENs and 22 “non-NENs” (tumors with an initial suspicion of NEN), were informatively stained for ISL1, and subsets also for INSM1 and/or SECG (as they were implemented later) and compared with staining outcomes of the traditional neuroendocrine markers CGA and SYP (Table 1). Non-NENs were defined as tumors in which a NEN diagnosis could be suspected based on histological features and therefore included in the study as controls. Positive neuroendocrine marker stainings (CGA/SYP) in non-NEN tumors were rarely encountered, and were almost always focal and occurred in 1–29% of the tumor cells only. As of this, all non-NENs contained cases in which the initial histology to a certain extent convinced the pathologist to submit slides for complementary IHC (including neuroendocrine markers)—and were therefore assumed to be credible controls mimicking the clinical difficulties in distinguishing these tumor entities from bona fide NENs. A single non-NEN in this cohort has been previously published [30].

**Table 1:** Summarized description of the Karolinska tumor cohort and immunohistochemical staining outcomes of informative cases

		Chromogranin A		Synaptophysin		ISL1		INSM1		Secretagogin	
Tumor type	No. of cases	Positive	Negative	Positive	Negative	Positive	Negative	Positive	Negative	Positive	Negative
Pancreatic NEN	32	30	2	31	0	30	2	22	0	8	2
Small intestinal NEN	25	25	0	24	0	0	25	16	1	15	2
NEN CUP	20	16	4	20	0	12	8	6	3	4	3
Colorectal NEN	14	10	4	14	0	11	3	8	1	7	0
Pheo/paraganglioma	9	9	0	9	0	9	0	7	0	1	5
Appendiceal NEN	6	6	0	6	0	1	5	5	0	5	0
Merkel cell carcinoma	4	3	0	4	0	4	0	1	0	1	0
Duodenal NEN	4	4	0	3	0	4	0	3	0	2	1
Gastric NEN	3	2	1	3	0	3	0	0	1	Nd	Nd
Lung NEN	3	3	0	3	0	3	0	2	0	2	0
Esophageal NEN	2	2	0	2	0	1	1	0	1	0	1
NAME	2	0	2	2	0	2	0	2	0	Nd	Nd
Cervical NEN	2	0	2	2	0	2	0	Nd	Nd	Nd	Nd
Renal NEN	2	2	0	2	0	2	0	1	0	1	0
Prostatic NEN	2	2	0	1	0	1	1	Nd	Nd	Nd	Nd
Parathyroid lesions	2	Nd	Nd	Nd	Nd	0	2	0	2	0	1
NEN in teratoma	2	2	0	2	0	2	0	Nd	Nd	Nd	Nd
MTC	2	2	0	1	0	2	0	2	0	2	0
Pancreatic MiNEN	1	1	0	1	0	1	0	Nd	Nd	Nd	Nd
Urinary bladder NEN	1	1	0	1	0	1	0	Nd	Nd	Nd	Nd
Olfactory neuroblastoma	1	1	0	1	0	0	1	1	0	1	0
Non-NEN*	22	3	17	8	11	6	16	2	5	0	5

*NEN*, neuroendocrine neoplasia; *CUP*, cancer of unknown primary; *pheo*, pheochromocytoma; *NAME*, neuroendocrine adenoma of the middle ear; *MTC*, medullary thyroid carcinoma; *MiNEN*, mixed neuroendocrine non-neuroendocrine neoplasia; *Positive*, focal or diffuse staining; *Negative*, absence of staining; *Nd*, not determined

*Non-NENs were initially suspected to be NEN based on histological features and therefore included in the study as controls. Positive staining in these tumors was almost always focal and occurred in 1–29% of tumor cells

Of all tumor cases in this study, 76 were stained as part of the primary diagnostics at our department and 85 were stained as part of the second opinion consultation workup of cases originally biopsied/operated outside of our department. The majority of the 161 tumors have been previously diagnosed by the same authors (CCJ and AH), and the few cases that were originally seen by another pathologist were subsequently re-analyzed histologically by CCJ before study inclusion.

### Test of ISL1, INSM1, and SECG Antigenicity Followed Prolonged Fixation

The material in this study is partly derived from multiple pathology centers around Sweden as second opinion referrals to our department. As the individual pathology units might exhibit local variations in the sample acquisition process (not least the fixation time; i.e., time from formalin fixation to the specimen is handled at the grossing station), we wanted to assess whether the immunoreactivity of the second-generation markers would diminish with prolonged fixation in formalin. As of this, pancreatic tissue samples of similar sizes from a de-identified patient were put in formalin and extracted for paraffin-embedment at 1 day, 3 days, 1 week, and 2 weeks after the start of fixation. The tissue samples were then re-embedded into the same paraffin block, subsequently cut and stained for ISL1, INSM1, and SECG, and evaluated for immunoreactivity using conventional light microscopy.

### Immunohistochemistry

All immunohistochemical analyses were performed using a clinically accredited platform (Ventana Medical Systems, AZ, USA) in a routine pathology laboratory setting. Four-micrometer sections from each tissue sample were de-paraffinized using xylen and ethanol and subsequently stained using the established protocol for each antibody. Primary antibodies used were anti-CGA (mouse monoclonal, clone LK2H10, Ventana, ready-to-use dilution), anti-SYP (rabbit monoclonal, clone SP11, Ventana, ready-to-use dilution), anti-Ki-67 (rabbit monoclonal, clone 30-9, Ventana, ready-to-use dilution), anti-ISL1 (rabbit monoclonal, clone EP283, Cell Marque, CA, USA, dilution 1:50), anti-INSM1 (mouse monoclonal, clone A-8, Santa Cruz Biotechnology, TX, USA, dilution 1:25), and anti-SECG (mouse monoclonal, clone 778518, R&D Systems, MN, USA, dilution 1:500). Antigen retrieval was performed using the Ventana Ultra CC1 buffer for all primary antibodies except for anti-SECG, for which the Ventana Ultra CC2 buffer was used. External positive controls consisted of normal pancreatic tissue (islets of Langerhans) and colonic mucosa (scattered neuroendocrine cells) and external negative controls consisted of a lymph node and normal kidney tissue. All controls were mounted on the same slide as the tissue of interest, and no slides without an expected signal in the external positive controls were included in the study. Expression was considered positive if > 90% of tumor cells were stained, focally positive if > 30% of tumor cells were stained for NEN cases and between 1 and 29% for non-NEN cases (as > 30% stained cells would change a diagnosis of non-NEN to a MiNEN or potentially also NEN). Negative staining was defined as < 1% tumor cells stained. For CGA and SYP, cytoplasmic staining was scored. For ISL1 and INSM1, nuclear staining was considered. For SECG, cytoplasmic and nuclear staining combined was considered.

### Statistical Analyses

Non-normal distribution was assumed for all data, applying Fisher’s exact test for comparison between groups. *P* values < 0.05 were considered as statistically significant. All calculations were prepared in SPSS version 25 (IBM SPSS Statistics, IBM, Armonk, New York, USA).

## Results

### First-generation Neuroendocrine Marker Staining Outcomes

The overall staining outcomes for each marker are summarized in Tables 1 and 2, and the individual staining results are presented in Supplementary Table 1. Out of the 139 NENs employed in this study, 136 were successfully assessed for CGA and 132 for SYP immunoreactivity. All observed stainings were cytoplasmic, as expected. For CGA, a total of 121 cases stained diffusely or focally positive, while 15 cases stained negative. Of these 15 cases, a total of 9 were pancreatic, cervical, gastric, and CUP neuroendocrine carcinomas (NECs, an entity known to dedifferentiate and lose CGA immunoreactivity), 4 were WHO grade I colorectal neuroendocrine tumors (NETs; known to frequently lack CGA immunoreactivity), and two were neuroendocrine adenomas of the middle ear (NAMEs). Of the 20 non-NENs investigated for CGA expression, 17 were negative and 3 found focally positive. For SYP, 132 NENs were positively stained, while 8 out of the 19 interrogated non-NENs showed SYP immunoreactivity. As expected, CGA and SYP immunoreactivity was significantly associated to a NEN diagnosis compared with that to non-NENs (Fisher’s exact test *P* < 0.0001 for both markers, Table 2) and displayed high sensitivity (CGA; 89%, SYP; 100%) and moderate to high specificity (CGA; 85%, SYP; 58%, Table 3) for the proper detection of NENs in a clinical routine material. The ensuing positive predictive value (PPV) was 98% (CGA) and 94% (SYP), and the negative predictive value (NPV) was 53% (CGA) and 100% (SYP) respectively.

**Table 2:** Immunohistochemical staining results of neuroendocrine markers among neuroendocrine and non-neuroendocrine neoplasms

	*IHC staining outcome*			*IHC staining outcome*			
**Chromogranin A (CGA)**	Positive/Focally positive	Negative	Fisher's Exact *P*	**Synaptophysin (SYP)**	Positive/Focally positive	Negative	Fisher's Exact *P*
**NENs** (n=136)	121	15	<0.0001*	**NENs** (n=132)	132	0	<0.0001*
**Non-NENs** (n=20)	3	17		**Non-NENs** (n=19)	8	11	
	*IHC staining outcome*			*IHC staining outcome*			
**ISLET1 (ISL1)**	Positive/Focally positive	Negative	Fisher's Exact *P*	**INSM1**	Positive/Focally positive	Negative	Fisher's Exact *P*
**NENs** (n=139)	91	48	0.0009*	**NENs** (n=85)	76	9	0.0007*
**Non-NENs** (n=22)	6	16		**Non-NENs** (n=7)	2	5	
	*IHC staining outcome*						
**Secretagogin (SECG)**	Positive/Focally positive	Negative	Fisher's Exact *P*				
**NENs** (n=64)	49	15	0.0014*				
**Non-NENs** (n=5)	0	5					

*IHC*, immunohistochemistry; *NENs*, neuroendocrine neoplasms; *Non-NENs*, non-neuroendocrine neoplasms

*Fisher’s exact P considered significant at *P* < 0.05

**Table 3:** Sensitivity, specificity, and predictive values of the neuroendocrine markers to distinguish NENs from non-NENs

IHC marker	Sensitivity	Specificity	Positive predictive value	Negative predictive value
Chromogranin A	89%	85%	98%	53%
Synaptophysin	100%	58%	94%	100%
ISL1	65%	73%	94%	25%
INSM1	89%	71%	97%	36%
Secretagogin	77%	100%	100%	25%

*IHC*, immunohistochemistry; *NEN*, neuroendocrine neoplasia; *non-NEN*, non-neuroendocrine neoplasia

### Discriminative Properties of Second-generation Markers Between NEN and Non-NEN Groups

**Fig. 1:**
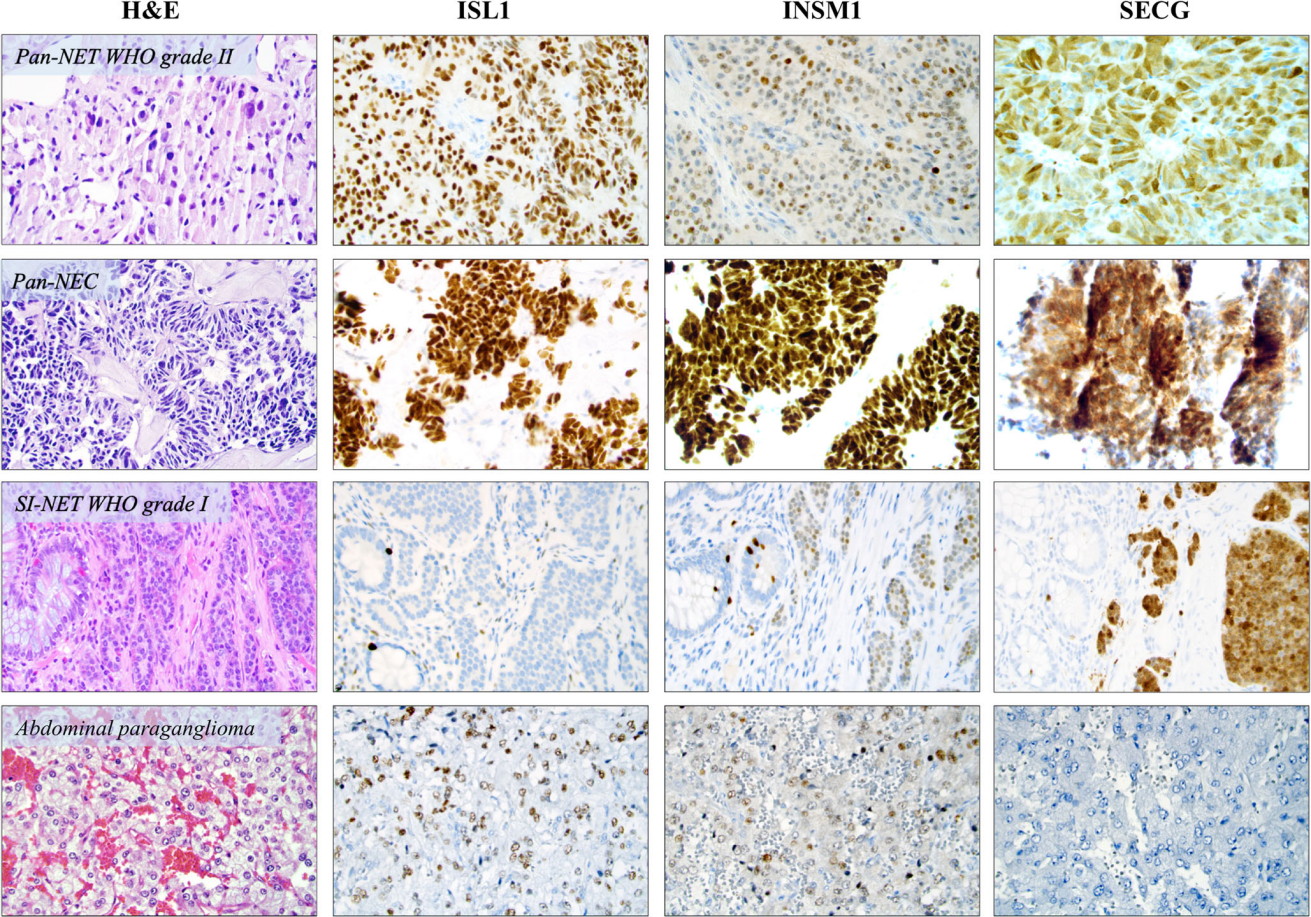
Immunoreactivity patterns of second-generation neuroendocrine markers in neuroendocrine neoplasms (NENs). All photomicrographs are magnified × 400. First column are routine-processed slides stained with hematoxylin-eosin (H&E), followed by columns displaying immunoreactivity patterns for ISL1, INSM1, and secretagogin (SECG). The first row illustrates a surgically resected primary grade II Pan-NET (pancreatic neuroendocrine tumor) exhibiting positive ISL1, focally positive INSM1 (mixture of negative and positive nuclei), and positive SECG expression. The second row depicts a liver metastasis core needle biopsy of a Pan-NEC (pancreatic neuroendocrine carcinoma). Note the consistent immunoreactivity for all three markers. The third row exemplifies the typical staining patterns of a grade I small intestinal NET (SI-NET) with negative immunoreactivity towards ISL1, and focal INSM1 expression adjoined by a positive SECG staining. Note the normal intestinal mucosal layer to the left of each image, with scattered, normal neuroendocrine cells as internal positive controls. The fourth row illustrates the recurrent staining pattern in pheochromocytomas and paragangliomas, exemplified here by an abdominal paraganglioma with focal positivity for ISL1 and INSM1, while SECG was negative

Diffuse or focal positive nuclear ISL1 immunoreactivity was noted in 91/139 NENs (65%) and in 6/22 (27%) non-NENs with an initial suspicion of neuroendocrine differentiation, and the difference between groups was strongly significant (*P* = 0.0009, Fisher’s exact test, Fig. 1, Table 2). No case with cytoplasmic ISL1 staining was found. The sensitivity for ISL1 to detect a NEN was 65%; the specificity was 73%, yielding a PPV of 94% and an NPV of 25%—suggesting that a positive ISL1 staining is strongly indicative of a NEN in the clinical setting when assessing tumors with histological suspicion of neuroendocrine differentiation, while a negative staining does not exclude the possibility of a NEN (Table 3).

INSM1 was noted with diffuse or focal nuclear positive immunoreactivity in 76/85 NENs (89%) and in 2/7 (29%) non-NENs with an initial suspicion of neuroendocrine differentiation (*P* = 0.0007, Fisher’s exact test, Fig. 1, Table 2). No case with cytoplasmic staining was observed. The INSM1 sensitivity for NENs was 89%; the specificity was 71%, with subsequent PPV and NPV values of 97% and 36% respectively (Table 3). In this regard, a positive INSM1 stain was even more indicative of a NEN than ISL1 positivity.

SECG was found diffusely or focally positive in 49 out of 64 assessed NENs (77%), while only 5 non-NENs were informatively stained, all with negative results (0%) (Fig. 1, Table 2). The sensitivity was 77% and the specificity 100%, leading to PPV and NPV values of 100% and 25% respectively (Table 3). The staining pattern was both cytoplasmic and nuclear for all cases, and no case with isolated cytoplasmic or nuclear immunoreactivity was noted.

### Absence of Correlation Between Immunoreactivity, Tumor Source, and WHO Grade

In total, we enlisted 43 NECs irrespectively of anatomical site. All informatively stained NECs were positive for SYP, whereas ISL1 positivity was noted in 86% of cases, INSM1 in 80%, SECG in 56%, and CGA in 81%, although the NECs stained for INSM1 and SECG were too few (*n* = 20 and *n* = 9 respectively) to draw meaningful conclusions (Supplementary Table 2).

For the two largest NEN groups in our clinical material, Pan-NENs (*n* = 32) and SI-NENs (*n* = 25), the amount of ISL1 stained cases was large enough to make meaningful assumptions regarding the potential association between staining outcome, tumor grade, and tumor property (primary tumor or metastasis). The Pan-NENs in this study were NET WHO grade I (*n* = 5), NET grade II (*n* = 12), NET grade III (*n* = 3), and NEC (*n* = 12), and a total of 26 out of the 32 cases (81%) were metastatic deposits, whereas the remaining samples were primary tumors (Supplementary Table 1). ISL1 immunoreactivity was noted in 5/5 grade I NETs, in 11/12 grade II NETs, in 2/3 grade III NETs, and in 12/12 NECs. The only two Pan-NENs with negative ISL1 were a primary grade II NET and a liver metastasis of a grade III NET. Therefore, the ISL1 expression was consistent throughout this Pan-NEN cohort irrespectively of tumor localization and WHO grade. For the SI-NENs, 14 cases were grade I NETs, while 11 were grade II NETs. Moreover, 16 samples were metastatic lesions (64%) and the remainders were primary tumors (Supplementary Table 1). As all samples (*n* = 25) were ISL1 negative, there was no correlation to either the tumor source or tumor grade of the SI-NEN cohort either.

### Tissue-Specific Expression Patterns

Among the 32 Pan-NENs, all informative tumors (31/31; 100%) displayed SYP positivity (diffuse or focal), and 30 out of 32 cases (94%) displayed CGA immunoreactivity (Table 1). A total of 30 out of 32 cases (94%) were diffusely or focally positive for ISL1 and all informative cases (22/22; 100%) stained diffusely or focally positive for INSM1. Moreover, SECG immunoreactivity was noted in 8 out of 10 (80%) informatively stained Pan-NENs.

All 25 SI-NENs included in this study stained unequivocally negative for ISL1 and were incontestably positive for CGA (25/25; 100%) and SYP (24/24 informative cases; 100%). In addition, 16 out of 17 informative SI-NEN cases (94%) were also diffusely or focally positive for INSM1 as well as 15 out of 17 (88%) for SECG. The same trend was noted for appendiceal NENs (*n* = 6), in which only one case was ISL1 positive (1/6; 17%), while cases were consistently positive for CGA (6/6; 100%), SYP (6/6; 100%), INSM1 (5/5; 100%), and SECG (5/5; 100%).

For NENs arising in the colo-rectum (*n* = 14; 11 rectal NENs and 3 colonic NENs), we found diffuse or focal positivity for ISL1 in 11/14 cases (79%), for INSM1 in 8/9 (89%) interrogated cases, and for SECG in 7/7 (100%) informative cases. As expected, while all 14 cases were diffusely or focally positive for SYP, 4/14 cases displayed negative CGA immunoreactivity.

### Associations Between the Expression of First- and Second-generation Neuroendocrine Markers

The majority of NENs with positive ISL1, INSM1, and/or SECG expression also exhibited positive expression when assessing markers of the first generation, although subsets of cases positive for second-generation markers were CGA-negative (Supplementary Table 3). On the other hand, SYP immunoreactivity was always noted in cases with positivity for ISL1, INSM1, and/or SECG, even in high-grade NECs.

### Test of ISL1, INSM1, and SECG Antigenicity

**Fig. 2:**
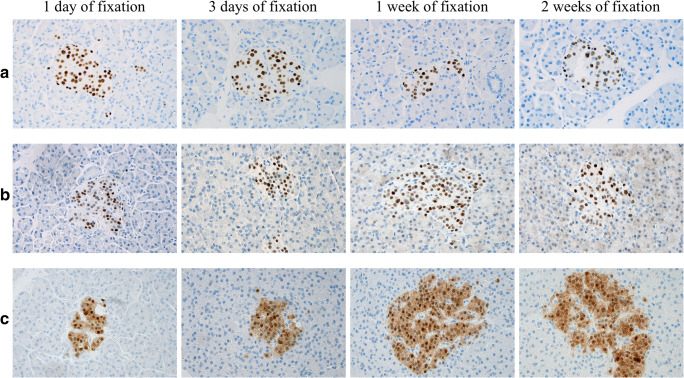
Assessment of ISL1, INSM1, and SECG antigenicity in normal pancreatic tissue. Normal pancreatic tissue samples of similar sizes were put in formalin and extracted for paraffin-embedment at 1 day, 3 days, 1 week, and 2 weeks after the start of fixation respectively. All photomicrographs display an islet of Langerhans exhibiting (**a**) diffuse nuclear ISL1 staining, (b) diffuse nuclear INSM1 staining, and (**c**) diffuse nuclear and cytoplasmic SECG immunoreactivity. The surrounding exocrine cells were consistently negative, besides a weak, cytoplasmic background signal appearing when staining for INSM1 after 2 weeks of fixation. These results indicate that negative immunohistochemical findings in our tumor cohort are not a consequence of poor antigenicity or prolonged tissue fixation. All photomicrographs are magnified × 400

As outlined in Fig. 2, the antigenicity for ISL1, INSM1, and SECG was not affected by prolonged formalin fixation in terms of staining intensity and the overall amount of Langerhans islet cells stained. Indeed, retained expression for all three markers was noted even after 2 weeks of fixation. In terms of specificity, the exocrine pancreatic tissue was negative, with the exception for a weak, cytoplasmic signal in the exocrine pancreatic tissue component when staining for INSM1 after 2 weeks of fixation.

## Discussion

**Fig. 3:**
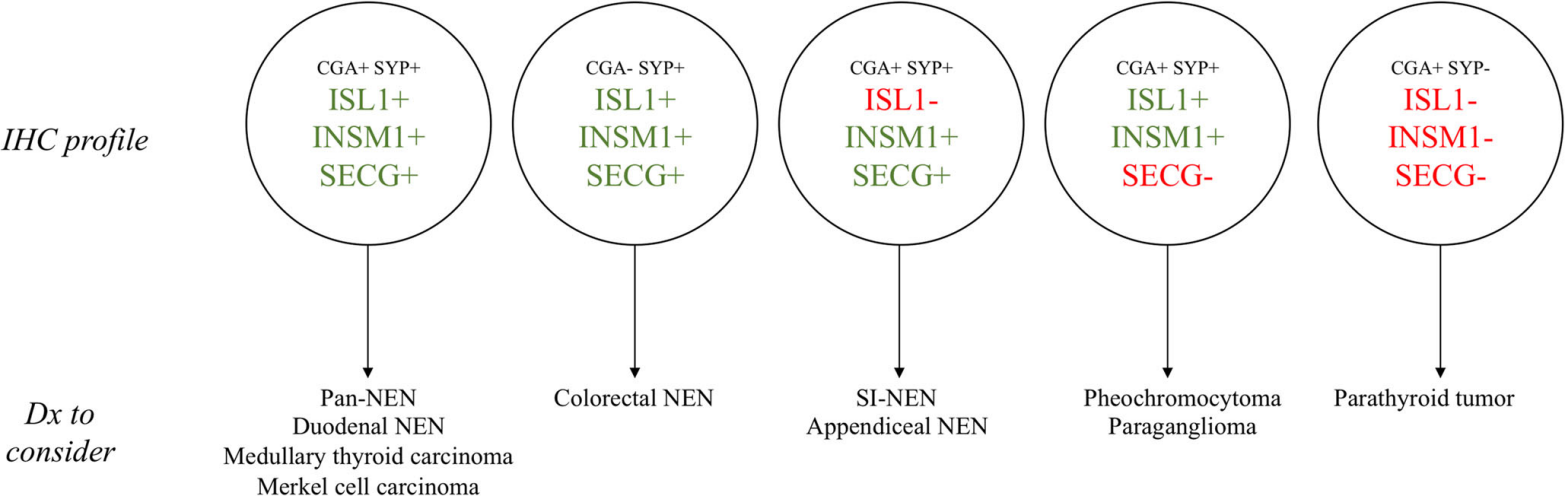
Schematic overview of the general second-generation neuroendocrine marker profiles and a suggested way of thinking regarding potential staining outcomes in metastatic NENs with an unknown primary. Most notably, NENs with immunoreactivity towards all three neuroendocrine markers of the second generation could constitute tumors with a wide variety of origin, whereas the triad ISL−, INSM1+, and SECG+ could indicate an origin in the small intestine or appendix. Additional tumor-specific markers not included in this study (serotonin, CDX2) could help verify this. A tumor exhibiting ISL1+, INSM1+, and SECG− should raise the suspicion of a pheochromocytoma or paraganglioma, and supplementary markers (GATA3, S100) are recommended. Please note that this scheme does not account for MiNEN and non-NENs with focal neuroendocrine differentiation, as these diagnoses are dependent on the proportion of tumor cells with neuroendocrine differentiation and hence could be differential diagnoses for tumors with all the abovementioned staining patterns. IHC, immunohistochemistry; +, positive or focal positive immunoreactivity; −, negative immunoreactivity; dx; differential diagnoses

We present our institutional outcome of clinical routine staining for neuroendocrine markers of both first- (CGA, SYP) and second-generations (ISL1, INSM1, SECG), and verify previously suggested immunophenotypic patterns of clinical use. As our material rely solely on a prospectively stained material in which all markers were investigated as part of the routine clinical workup, we here conclude that the markers have adequate sensitivity and specificity when put against tumors with an initial histological suspicion of neuroendocrine differentiation, but in the end not fulfilling the criteria for a bona fide NEN. We also propose a comprehensive scheme regarding tissue-specific immunohistochemical profiles for diagnostic purposes, and suggest that ISL1, INSM1, and SECG can aid in the proper identification of the primary site of a metastatic NEN with unknown origin (Fig. 3).

In general, all three markers of the second generation seem to display high sensitivity for NENs, despite with some tissue-specific profiles (discussed below). ISL1, INSM1, and SECG all presented with exceedingly high PPVs concerning the distinction between NENs and non-NENs in the clinical setting, irrespective of WHO grade or tumor origin (primary tumor vs. metastasis). Hence, a focally positive or positive ISL1, INSM1, and/or SECG staining (in > 30% of cells, which was the cut-off for focal positivity in the NEN group) is highly indicative of a NEN when the pathologist is facing a non-NEN tumor with a histological suspicion of a NEN. Regarding the specificity of the markers in our series, SECG stood out as the most reliable performer in this aspect. Although only few of our institutional non-NENs were stained for this marker, our data could suggest that the excellent SECG specificity makes it a good candidate for clinical tumoral investigations in which a neuroendocrine differentiation is suspected.

From a clinical standpoint, one of the most pressing matters is to identify immunohistochemical markers with superior sensitivity compared with the expression of traditional markers of the first generation, which are recurrently reduced or lost in high-grade NECs. In our material, we verify that ISL1 is a sensitive marker also for the identification of NECs with an overall sensitivity of 86%. Although this was lower than the sensitivity for the first-generation marker SYP (100%), one should note that several of the ISL1 negative NECs cases in our series were derived from the lower GI tract (an anatomical region in which NENs recurrently are reported absent for ISL1 expression). Overall, the majority of NENs (including NECs) with positive ISL1, INSM1, or SECG expression were also positive for both CGA and SYP, but subsets of these NENs were CGA-negative while consistently SYP-positive—vaguely suggesting that a combination of SYP adjoined by second-generation neuroendocrine markers could constitute a highly sensitive and specific panel for clinical usage (Supplementary Table 3).

Several tissue-specific patterns were recognized in this study, of which some have been proposed in previous publications. For example, positivity for CGA, SYP, ISL1, INSM1, and SECG was a common profile for Pan-NENs irrespectively of tumor grade or tumor site (primary lesion vs. metastatic lesion), while an immunohistochemical profile consisting of a negative ISL1 staining adjoined by positivity for other neuroendocrine markers of both the first- and second-generation would strongly suggest an origin in the small intestine or appendix. Indeed, previous studies have pinpointed high sensitivity for ISL1 and INSM1 immunoreactivity in Pan-NENs, as well as widespread ISL1 negativity in SI-NENs [6, 8, 31–33].

A few previously uncharacterized profiles of potential value for diagnostic purposes were also evident. For colorectal NENs, positivity for ISL1, INSM1, SECG, and SYP was a consistent profile, even in the absence of CGA immunoreactivity—the latter phenomenon not being uncommon for NENs arising in the large intestine (Table 1, Fig. 3). To our knowledge, INSM1 and SECG immunoreactivity has not yet been thoroughly assessed in colorectal NENs apart from single observations in limited case series [26, 34]. Moreover, rare cases of renal NENs and NENs arising in teratomas included in our study were consistently positive for ISL1, which are novel findings worth exploring in larger materials.

For PPGLs, a profile with positive ISL1 and INSM1 stainings adjoined by negative immunoreactivity for SCG was commonly observed (Table 1, Fig. 3). These results are somewhat contradictory from previous findings suggesting widespread SCG positivity in NENs from the adrenal glands [24, 26, 27]. However, we validated our findings by also scrutinizing the immunoreactivity in adjacent, histologically normal adrenal glands from the same patients (Supplementary Fig. 1). We can only speculate regarding the underlying reasons for these methodological discrepancies, but previous studies were either conducted using northern blot analyses [24] or with immunohistochemistry using polyclonal antibodies for the majority of analyses [26, 27], whereas we employed a monoclonal SCG antibody. Future studies regarding SCG expression in normal adrenal medulla and PPGLs are therefore highly warranted, as metastatic PPGLs can be hard to pinpoint, as established clinical markers (such as GATA3 and S100) not always stain positive, making an auxiliary high-sensitivity marker of imminent clinical value.

This study mainly revolves around ISL1 immunohistochemistry, which is due to the fact that the marker was introduced in our clinical screening well before INSM1 and SECG. As the aim of this study was to report the clinical utility of all these markers, there is a predominance of ISL1 stained tumors that often lacked stains of the two other markers. As tumors were not stained in retrospect, we recognize that our conclusions regarding INSM1 and SECG immunoreactivity as discriminative markers should be interpreted with caution and our findings reproduced in larger materials. Moreover, many of our NEN subgroups are rare manifestations of NENs not commonly encountered in clinical practice, for example renal, esophageal and cervical NENs as well as NENs in teratomas. Although our ISL1, INSM1, and SECG data regarding many of these NEN subtypes are novel and therefore warrant attention, we lack power to safely assess the immunohistochemical profiles of these extraordinary NEN subtypes from a diagnostic standpoint.

To summarize, we verify previous observations that immunohistochemical staining using neuroendocrine markers of the second generation should act as a complement to the established markers CGA and SYP and recommend these markers to be implemented in routine clinical practice.

## Electronic supplementary material

ESM 1 (PNG 6376 kb)

High Resolution Image (TIFF 42385 kb)

ESM 2 (XLSX 35 kb)

ESM 3 (XLSX 10 kb)

ESM 4 (XLSX 44 kb)
