# Supplementary figures and images for: Clinical Routine Application of the Second-generation Neuroendocrine Markers ISL1, INSM1, and Secretagogin in Neuroendocrine Neoplasia: Staining Outcomes and Potential Clues for Determining Tumor Origin
Source: Endocr Pathol. 2020 Aug 19;31(4):401–10. doi: 10.1007/s12022-020-09645-y (PMC7665972; doi:10.1007/s12022-020-09645-y)

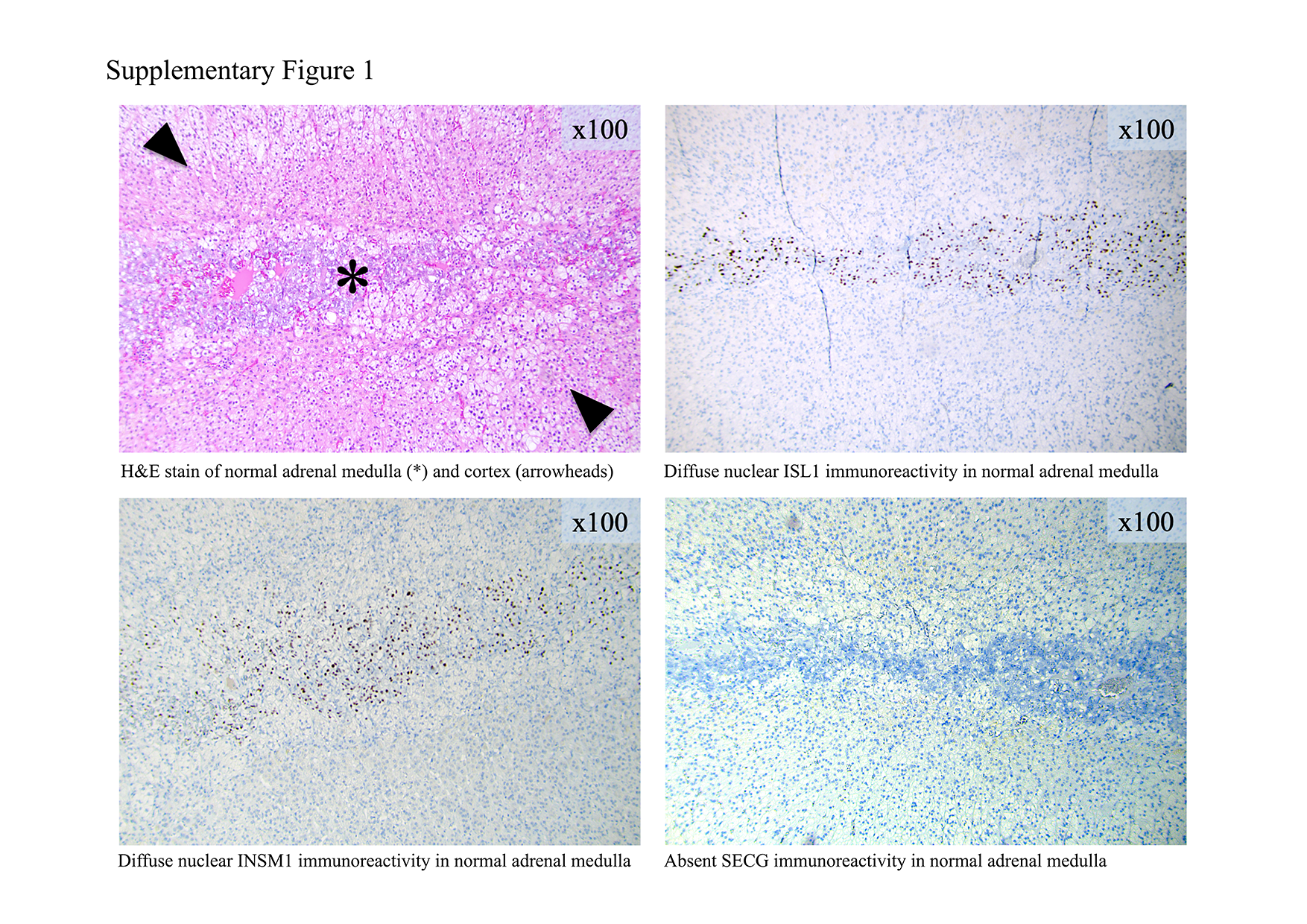

Supplement: Supplementary file 1 — (PNG 6376 kb) [file 12022_2020_9645_Fig4_ESM.png]

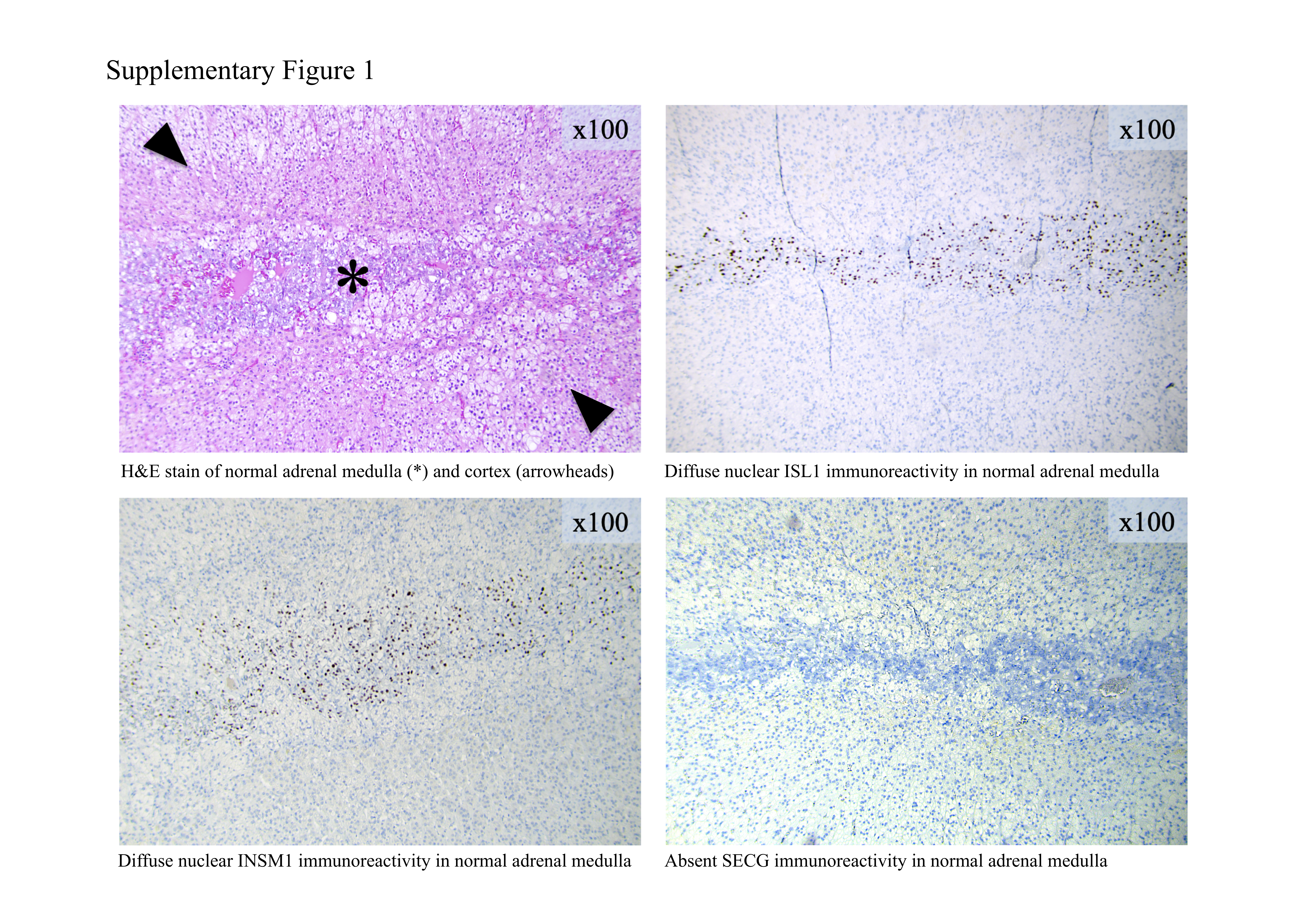

Supplement: Supplementary file 2 — High Resolution Image (TIFF 42385 kb) [file 12022_2020_9645_MOESM1_ESM.tiff]
